# Co-design and prototype development of MedManageSCI: a medication self-management toolkit for adults with spinal cord injury/dysfunction

**DOI:** 10.1186/s12913-025-12705-w

**Published:** 2025-04-22

**Authors:** Lauren Cadel, Rasha El-Kotob, Sander L. Hitzig, Lisa M. McCarthy, Shoshana Hahn-Goldberg, Tanya L. Packer, Tejal Patel, Stephanie R. Cimino, Aisha K. Lofters, Chester H. Ho, Maliha Asif, Sara J.T. Guilcher

**Affiliations:** 1 Leslie Dan Faculty of Pharmacy, University of Toronto, Toronto, ON Canada; 2 Institute for Better Health, Trillium Health Partners, Mississauga, ON Canada; 3 St. John’s Rehab Research Program, Sunnybrook Research Institute, Sunnybrook Health Sciences Centre, Toronto, ON Canada; 4 Rehabilitation Sciences Institute, Temerty Faculty of Medicine, University of Toronto, Toronto, ON Canada; 5 Department of Occupational Science and Occupational Therapy, Temerty Faculty of Medicine, University of Toronto, Toronto, ON Canada; 6 Dalla Lana School of Public Health, University of Toronto, Toronto, ON Canada; 7 School of Pharmacy, University of Waterloo, Kitchener, ON Canada; 8 Department of Family and Community Medicine, University of Toronto, Toronto, ON Canada; 9 Women’s College Research Institute, Toronto, ON Canada; 10 Openlab, University Health Network, Toronto, ON Canada; 11 Schools of Occupational Therapy and Health Administration, Dalhousie University, Halifax, NS Canada; 12 Department of Nursing, Umeå University, Umeå, Sweden; 13 Schlegel-University of Waterloo Research Institute of Aging, Waterloo, ON Canada; 14 Lawson Research Institute, St. Joseph’s Healthcare, London, ON Canada; 15 Division of Physical Medicine & Rehabilitation, Department of Medicine, University of Alberta, Edmonton, AB Canada; 16 Institute of Health Policy, Management and Evaluation, University of Toronto, Toronto, ON Canada; 17 Department of Physical Therapy, Temerty Faculty of Medicine, University of Toronto, Toronto, ON Canada

**Keywords:** Spinal cord injury, Spinal cord dysfunction, Medication self-management, Co-design, Toolkit, Self-management, Digital health intervention, Medication adherence, Self-care, Participatory research, Chronic condition, User-centered innovation

## Abstract

**Background:**

Medications are among the most common health interventions, with certain populations, such as individuals with spinal cord injury/dysfunction (SCI/D), commonly prescribed multiple medications. Consequently, adults with SCI/D often engage in activities related to medication self-management, but there are few comprehensive resources for this population. The objective of this study was to co-design the prototype of a toolkit to support medication self-management among adults with SCI/D.

**Methods:**

We conducted a participatory multi-methods study, using the Good Things Foundation Pathfinder Model as a guide for the co-design process. Participants included adults with SCI/D, caregivers, and healthcare providers. Following the model’s three stages, we: (1) understood and defined the problem by conducting a scoping review, concept mapping study, and working group sessions; (2) created a prototype of the toolkit through working group sessions and website development meetings; and (3) tested the prototype through working group sessions.

**Results:**

The working group consisted of 19 individuals, including 9 adults with SCI/D, 1 caregiver, and 9 healthcare providers. In Stage 1, we identified the need for a comprehensive medication self-management resource through a scoping review, brainstormed content and delivery methods, and thematized and prioritized the content into eight categories through a concept mapping study. The concept mapping study included 44 participants, including 21 adults with SCI/D, 11 caregivers, and 12 healthcare providers. In Stage 2, feedback on the content mapped onto five categories: first impressions, message and purpose, visual elements, layout and flow, and graphics. The name, MedManageSCI, was selected by the working group. Through an iterative process with the website development company, an online version of the toolkit prototype was created (www.medmanagesci.ca). In Stage 3, participants provided recommendations to improve the website’s functionality and navigation.

**Conclusions:**

The co-design of the MedManageSCI prototype is a significant step toward addressing the medication self-management needs of adults with SCI/D. The implications of this work extend beyond SCI/D, highlighting the importance of tailored digital health resources for populations with complex healthcare needs. Future work is needed to refine the content, assess the feasibility, acceptability, and appropriateness of the toolkit, and examine outcomes related to medication self-management.

## Background

Medications are among the most common health-related therapeutic interventions [1], with surveys in Canada [2] and the United States [3] indicating that approximately 50% of participants had taken at least one prescription medication in the month prior to the study (2016–2019 and 2015–2018, respectively). Medication self-management can be defined as the everyday tasks, skills, and behaviours needed to manage the physical, social, and cognitive aspects of taking, or choosing not to take medications [4]. Aligned with Lorig and Holman’s [5] core tasks of self-management, medication self-management involves having the knowledge, confidence, and capabilities for medical, emotional, and role management, along with the core skills of problem-solving, seeking supports, making decisions, setting and tailoring goals, and engaging in activities and social interactions related to managing medications [4–6].

Individuals with chronic conditions are often prescribed medications and consequently, are self-managing their medications. While the management of medications occurs if an individual is taking one or multiple medications, the complexity of self-management is amplified, with increased tasks and skills required when managing multiple medications. For example, adults with spinal cord injury/dysfunction (SCI/D) are individuals who frequently engage in medication self-management as they often take multiple medications, with several studies noting a high prevalence of polypharmacy (often defined as the use of five or more medications) [7–13]. Following SCI/D, individuals can experience numerous secondary conditions due to changes in their autonomic, sensory, and motor functions caused by the damage to the spinal cord [14]. While many secondary conditions are experienced [15–19], pain, spasticity, constipation, urinary tract infections, sexual dysfunction, and pressure injuries are amongst the most common [19]. Secondary conditions may be acute, chronic, or episodic and individuals with SCI/D can experience multiple secondary conditions concurrently. Medication use generally increases post-SCI/D to support the management or treatment of these secondary conditions [12, 20]. This increase in medication use can lead to challenges across all areas of medication self-management. For instance, a qualitative study conducted by Cadel and colleagues exploring the lived experiences of medication management with persons with SCI/D identified challenges included limited understanding of one’s medication regimen, difficulty integrating medication into daily routines, uncertainty around managing side effects, hesitation when communicating with healthcare providers, and challenges dealing with fear of negative outcomes [21].

Given these challenges, paired with the limited availability of support tools, the development and implementation of educational material targeting medication self-management has been recommended for people with SCI/D [4, 21]. These recommendations are based on the previously described qualitative study by Cadel and colleagues [21]. A toolkit houses information aimed at educating a specific population, sharing knowledge, and promoting positive behaviour change [22]. While there is limited literature to draw on from the SCI/D population, medication self-management toolkits have revealed promising outcomes among other neurological populations including adults with multiple sclerosis [23]. In a scoping review conducted by Guilcher at al., four medication self-management toolkits were identified [23]. Three were technology-based (web or smartphone; MSmonitor, Web-based Intervention Support System, Smartphone-based Application), while one was paper-based (Relapse Management Course). Improvements in medication adherence (Web-based Intervention Support System), autonomous decision-making (Relapse Management Course), symptom management, (MSmonitor), self-management knowledge (MSmonitor), quality of life (MSmonitor), and quality of healthcare (MSmonitor and Web-based Intervention Support System) were identified following use of the toolkits.

The use of non-medication focused toolkits in the SCI/D population has demonstrated positive outcomes, including increased engagement in physical activity, and enhanced understanding and management of secondary conditions [24–27]. As such, there is potential to develop a comprehensive medication self-management toolkit for adults with SCI/D to address challenges previously reported. Involving end-users throughout this process is of key importance to ensure the toolkit is relevant and meets the needs of adults with SCI/D.

Co-design involves the active engagement of people with lived experience and other key interest groups in developing solutions to complex problems [28, 29]. Leveraging design-thinking principles along with individuals’ collective competence and creativity, this participatory approach ensures the relevance and applicability of the developed solution to the end-users [28, 29]. In an optimal co-design process, key interest groups are provided with agency in the process and their values and goals are respected [30, 31]. Other added benefits of co-design include increased adoptability and sustainability of the solution, an enhanced learning environment for researchers and key interest groups, and improved relationships between researchers and key interest groups [28, 29]. Given these benefits of co-design and the importance of medication self-management among this population, the objective of this study was to co-design the prototype of a toolkit to support medication self-management among adults with SCI/D.

## Methods

### Theoretical orientation

This research was grounded in pragmatism, which integrates assumptions from positivism and interpretivism [32, 33]. Pragmatism is linked to action and based on practical solutions. It acknowledges that knowledge is created and based on an individual’s experiences, including their social interactions. As researchers situated within the health services field, we acknowledge that the toolkit is a practical solution, and we will be able to identify if it works to improve medication self-management by assessing different outcomes.

### Study design

**Fig. 1:**
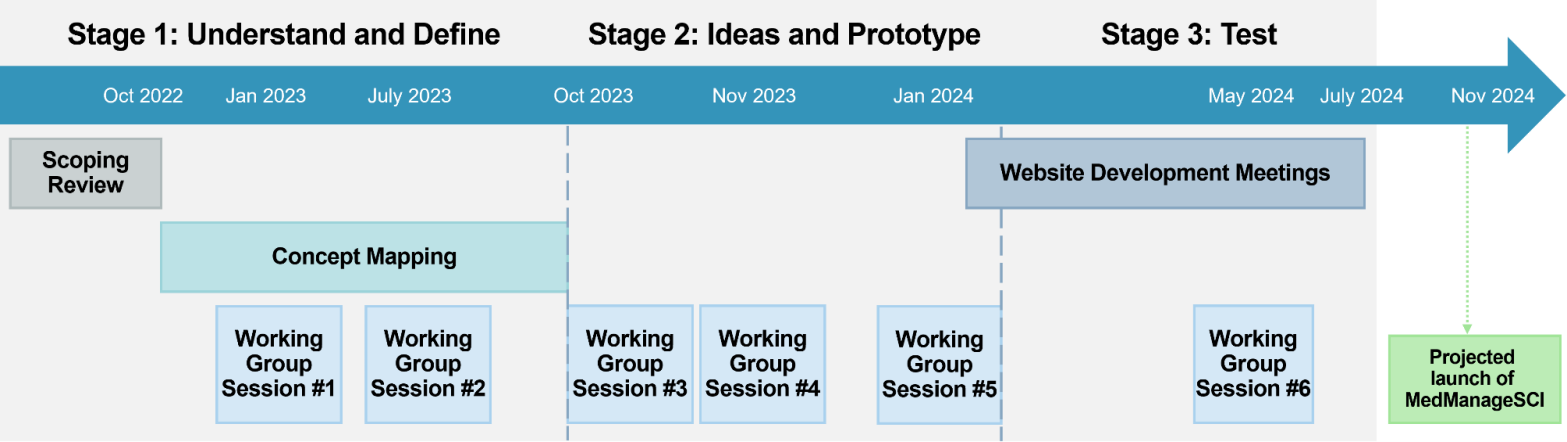
Overview of co-design stages, activities, and timelines

This was a participatory, multi-methods study leveraging principles of co-design. We used the Good Things Foundation Pathfinder Model [34] as the primary guide for the co-design process, but also drew on additional participatory methodologies [35, 36]. Following the three stages outlined in the Pathfinder model, we (1) understood and defined the problem by engaging with end-users and other key interest groups to learn about their needs; (2) created a prototype of *the thing* (toolkit) by learning about the end-users and other key interest groups’ ideas and preferences; and (3) tested the prototype by engaging with the end-users and other key interest groups to obtain their feedback (Fig. 1).

### Participant sampling and recruitment

Purposive and convenience sampling strategies were used to recruit participants beginning in October 2022. More specifically, participants from previous projects who had consented to be recontacted were informed of the study via email, flyers were posted on social media, and information was distributed by partner organizations and the research teams’ networks. To participate, individuals with SCI/D were required to have either a traumatic or non-traumatic injury, be at least three months post-injury, and live in Canada. Caregivers were required to provide care to support an adult with SCI/D who was at least three months post-injury and live in Canada. Healthcare providers were required to provide health or social services to adults with SCI/D and be employed in Canada. All participants had to be at least 18 years of age and be able to read and communicate in English. We sought variation in injury type, age, gender, sex, race, and healthcare provider profession; however, no participants were excluded based on these demographics. All interested individuals who met the eligibility criteria were included.

### Stage 1: understand and define

The purpose of Stage 1 was to understand end-user (adults with SCI/D) and other key interest groups’ needs (caregivers and healthcare providers). To do so, a scoping review was conducted first, followed by a concept mapping study and two working group sessions. Additional details of the scoping review [4] and concept mapping study [37] are published elsewhere, but summarized below.

#### Scoping review

Given that scoping reviews are well-situated to map the current state of the literature [38], the aim of the scoping review was to identify what was reported on medication self-management interventions for adults with SCI/D [4]. The scoping review was conducted to obtain a better understanding of relevant work that had already been done to potentially draw from or build on it. Conducting a scoping review also allowed for the identification of gaps in the literature [39].

#### Concept mapping

Next, concept mapping and working group sessions occurred concurrently. The aim of the concept mapping study was to identify content to include in the medication self-management toolkit, as well as considerations around the delivery of the toolkit [37]. This mixed methods approach involved both end-users and key interest groups, as participants consisted of adults with SCI/D, caregivers, and healthcare providers. Brainstorming sessions were conducted to generate ideas about the content to include in the toolkit through virtual focus groups or individual asynchronous sessions. These sessions were led by an experienced facilitator (LC), who used a structured script that was developed based on concepts from the Behaviour Change Wheel [40]. All statements generated were recorded and synthesized by the research team for subsequent tasks. During the sorting and rating stage, participants created thematic clusters of statements and rated each statement on how important and realistic it would be to include in the toolkit. These tasks were completed independently by participants using an online software. A sub-set of participants took part in the mapping session, which was conducted as a virtual focus group. During this session, a final concept map was selected by participants and thematic clusters of statements were finalized. All decisions were made through group discussion, led by a member of the research team (LC). The concept mapping activities occurred between October 2022 and October 2023. Results from these activities were used to inform the initial development and delivery of the toolkit.

#### Working group sessions

One working group, comprised of adults with SCI/D, caregivers, and healthcare providers, was formed to guide this study. Working group members were able to participate in the research activities if interested. All working group sessions were held virtually on Zoom and lasted between 60 and 90 min. The sessions were facilitated by a member of the research team (LC) and they were not audio or video recorded. A designated notetaker took detailed notes to capture the discussion, feedback, and questions. Each working group session had pre-established objectives that were shared with the group at the beginning of the session. Working group members could provide feedback and ask questions throughout the sessions, but each session also included structured discussion questions to gather specific input. The discussion questions were shared with members via screensharing on Zoom and through the chat function. Decisions regarding the toolkit prototype were achieved through group discussion and shared decision-making between the research team and the working group. Shared decision-making was a two-way, collaborative process through which the research team and working group engaged in meaningful conversation to interpret findings, improve the visual content and resources within the toolkit, and guide next steps. We experienced some situations where members of the working group had contrasting views and opinions with each other. In these situations, if consensus amongst the working group members could not be achieved through discussion, the research team would launch an anonymous poll on Zoom to finalize the decision. We did not have any situations where the research team and working group as a whole had opposing views. Engaging with the working group was an essential part of the toolkit prototype co-design because while the research team brought expertise in the methods, the working group brought lived experience and both contextual and practical knowledge that significantly enhanced the toolkit.

The first session was held in January 2023, with the purpose being to develop and build rapport among members, set expectations and goals for the working group, and reflect on preliminary concept mapping brainstorming sessions. Breakout rooms were used to facilitate discussion around the concept mapping brainstorming results. Specifically, working group members were guided through a discussion by an individual from the research team to understand their overall reactions to the findings and brainstorm ideas about how the content areas could be delivered. The second session was held in July 2023, with the purpose being to thematize and prioritize content ideas. The working group completed independent tasks (e.g., concept mapping sorting and rating) and engaged in a group discussion guided by a member of the research team. Through the concept mapping and working group sessions, consensus was achieved in how the toolkit would be delivered, via a website.

### Stage 2: ideas and prototype

#### Working group sessions

The purpose of Stage 2 was to identify *the thing*, a prototype of the toolkit, through content and website development. To do so, weekly team meetings, three working group sessions, and a series of website planning and development meetings were held. The core research team (LC, RE, MA, SJTG) developed written and visual content for the toolkit based on the findings from Stage 1. Content development was based on research evidence, guidelines, best practices, and lived experiences of persons with SCI/D. Written content was reviewed by members from the broader research team and experts in the relevant field (e.g., pharmacists, researchers, nurses, scientists).

The third working group session took place in October 2023 and the fourth was in November 2023. The purpose of these sessions was to obtain in-depth feedback on the visual content and resources being developed for the toolkit, such as: infographics, videos, and downloadable resources. As the targeted end-users of the toolkit, these sessions only consisted of adults with SCI/D. Both sessions were held on Zoom and were 60 min in length. The sessions were not recorded, but a notetaker was present to capture feedback.

The fifth working group session was held in January 2024. It involved all members of the working group. The purpose of this session was to present the concept mapping results, finalize the modules for the toolkit, decide on a name for the toolkit, and discuss design and brand considerations (e.g., colour scheme, fonts, vision). To decide on a name of the toolkit, the research team had a brainstorming meeting prior to the session to create five potential options to present to the working group. Breakout rooms were used to facilitate discussion. Each breakout room was facilitated by a member of the research team and included a designated notetaker. Following the breakout rooms, the working group debriefed about their conversations regarding the name of the toolkit and consensus was achieved.

#### Website development meetings

Website planning and development meetings occurred between February 2024 and July 2024. The core research team (LC, RE, SJTG) had weekly meetings with the website development company over this six-month period to achieve a number of aims, including: developing the brand identity (e.g., mission, vision, values, goals, attributes), identifying design features (e.g., colour scheme, fonts, logo, layout), ensuring accessibility (e.g., colour contrast, functionality), and developing desktop and mobile design mock-ups.

### Stage 3: test

#### Working group sessions

The purpose of Stage 3 was to revise and preliminarily test the prototype of the toolkit website by obtaining feedback. To do so, a sixth working group session was held on Zoom in May 2024. At this time, the website was password protected and not publicly available. The working group was guided through the website by a member of the research team and their feedback was sought on the following areas: layout, functionality, testimonials, and team biographies. The facilitator also inquired about their overall reactions and general recommendations for improvement. The working group’s feedback was recorded by a notetaker.

### Data analysis

Analyses of the scoping review and concept mapping study are published elsewhere [4, 37]; here, we report the analysis of the working group sessions. The notes from all working group sessions were compiled and discussed by the research team in subsequent meetings. The data were analyzed descriptively through inductive coding [41]. One member of the research team (LC) applied a word or short phrase to the detailed notes from the sessions. These words and short phrases were identified as the researcher read the data. The coded data were reviewed and organized based on conceptual similarities. This organization supported the combination and condensing of codes into categories, which were then labelled. Categorization, based on the types of revisions and feedback provided, was conducted by one member of the team (LC) and reviewed by a second (RE). Suggested revisions were discussed by the research team, along with specific actions to address them. Data were organized and analyzed using Microsoft Word and Microsoft Excel.

### Ethics

All participants provided written informed consent prior to participation. This study received ethics approval from the Research Ethics Boards of the University of Toronto (#42195) and the University of Alberta (#Pro00121103). All research was carried out in compliance with the Declaration of Helsinki.

## Results

### Stage 1: understand and define

The results of the scoping review and concept mapping study are briefly described below as they have been previously reported and are published elsewhere [4, 37].

#### Scoping review

The scoping review identified limited literature, as only three studies, with three unique interventions were included [4]. One of the interventions specifically targeted medication management, while the others included medication management as part of a larger intervention. While all interventions addressed some tasks and/or skills related to medication self-management, none did so in a comprehensive manner. One of the interventions was developed in consultation with patients, caregivers, and healthcare providers. In terms of results, there was minimal overlap in assessed outcomes, but some improvements were noted in clinical, learning, and behavioural outcomes. As such, a key recommendation from this scoping review was to co-design a resource for adults with SCI/D that comprehensively addresses and measures outcomes related to medication self-management [4]. 

#### Concept mapping

The concept mapping study included 44 participants, including 21 adults with SCI/D, 11 caregivers, and 12 healthcare providers (demographics reported elsewhere) [37]. The concept mapping activities identified a total of 79 statements, which were categorized into eight clusters: information-sharing and communication, healthcare provider interactions and involvement, peer and community connections, supports and services for accessing prescription medications and medication information, information on non-prescription medication and medication supplies, safety and lifestyle considerations, general medication information, and practical information and strategies related to medication-taking. Of these clusters, safety and lifestyle considerations was perceived by adults with SCI/D and healthcare providers to be the most important and realistic cluster to include in the toolkit. While all clusters were included in the toolkit, initial content development focused on those rated highest on importance and realistic [37]. 

#### Working group sessions

The working group consisted of a total of 19 individuals, including nine adults with SCI/D, one caregiver, and nine healthcare providers (the number of working group members who attended each session is presented in Table 1). Of all working group members, 15 identified as women and 4 identified as men. Nine members identified as white and 10 identified as other races (e.g., South Asian, East/Southeast Asian, Indigenous, mixed). Members were from two provinces in Canada, Ontario and Alberta. The healthcare providers were employed as pharmacists, physicians, occupational therapists, physical therapists, and nurses. Of those with SCI/D, six had a traumatic injury and three had a non-traumatic injury. Two individuals with SCI/D were taking between one and four medications per day, two were taking between five and nine medications, two were taking between ten and fourteen medications, and three were taking 15 or more medications.

**Table 1:** Breakdown of participant numbers by working group session

	Working Group Members		
	Adults with SCI/D	Caregivers	Healthcare providers
Working group meeting #1	6	1	5
Working group meeting #2	6	1	4
Working group meeting #3	6	Not applicable	Not applicable
Working group meeting #4	6	Not applicable	Not applicable
Working group meeting #5	6	0	4
Working group meeting #6	9	0	3

In reflecting on the initial results from the concept mapping study during the first session, members of the working group identified that the information was comprehensive, helpful, and easy to understand. Specific to information delivery, the working group discussed the importance of presenting content in a variety of ways, including text, videos, infographics, and pictures. They talked about an online toolkit being an ideal method of delivery, but also emphasized the importance of ensuring accessibility of the toolkit in terms of design and function (e.g., appropriate colour contrast, font size, clean layout, limited scrolling). For example, the working group members talked about their preference for a large font, having a balance between written and visual content with not too much text, and having solid colours in the headings of infographics, rather than gradient. Lastly, the working group wanted the toolkit to have a ‘catchy’ name that users would remember.

In the second working group session, individuals engaged in the sorting and rating activities from the concept mapping study [37] and reflected on the process. The results of the concept mapping sorting and rating activities are reported elsewhere [37], so here, we reflect on the participants’ feedback on the process of thematizing and prioritizing the content ideas. During the session, the working group was provided with approximately 40 min to complete the activities. Some individuals completed both activities within this allotted time, while others had to complete them on their own time. One member recommended providing the instructions ahead of time to allow for more time for the activities during the session. Individuals described the sorting task as more challenging to complete than the rating. Some additional recommendations provided by the working group included: asking individuals to complete the rating task first to familiarize themselves with the statements, allowing all participants to share their screens to help with troubleshooting potential issues, and ensuring all individuals have their videos off and sound muted for the duration of the activities to avoid distractions. Overall, working group members found the concept mapping software easy to use, which improved their experience.

### Stage 2: ideas and prototype

#### Working group sessions

**Fig. 2:**
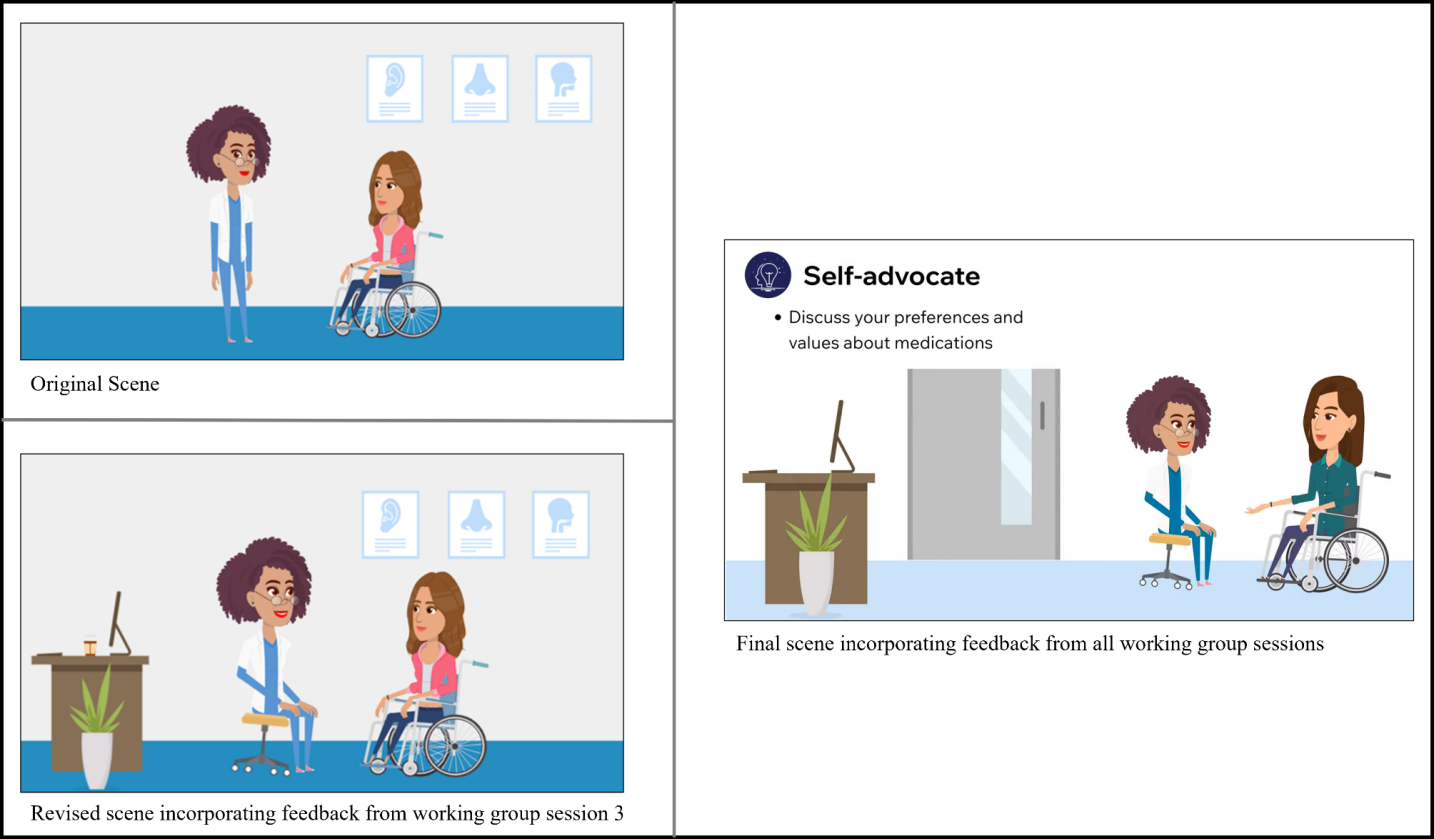
Original, revised, and final video scenes based on the working group sessions

**Fig. 3:**
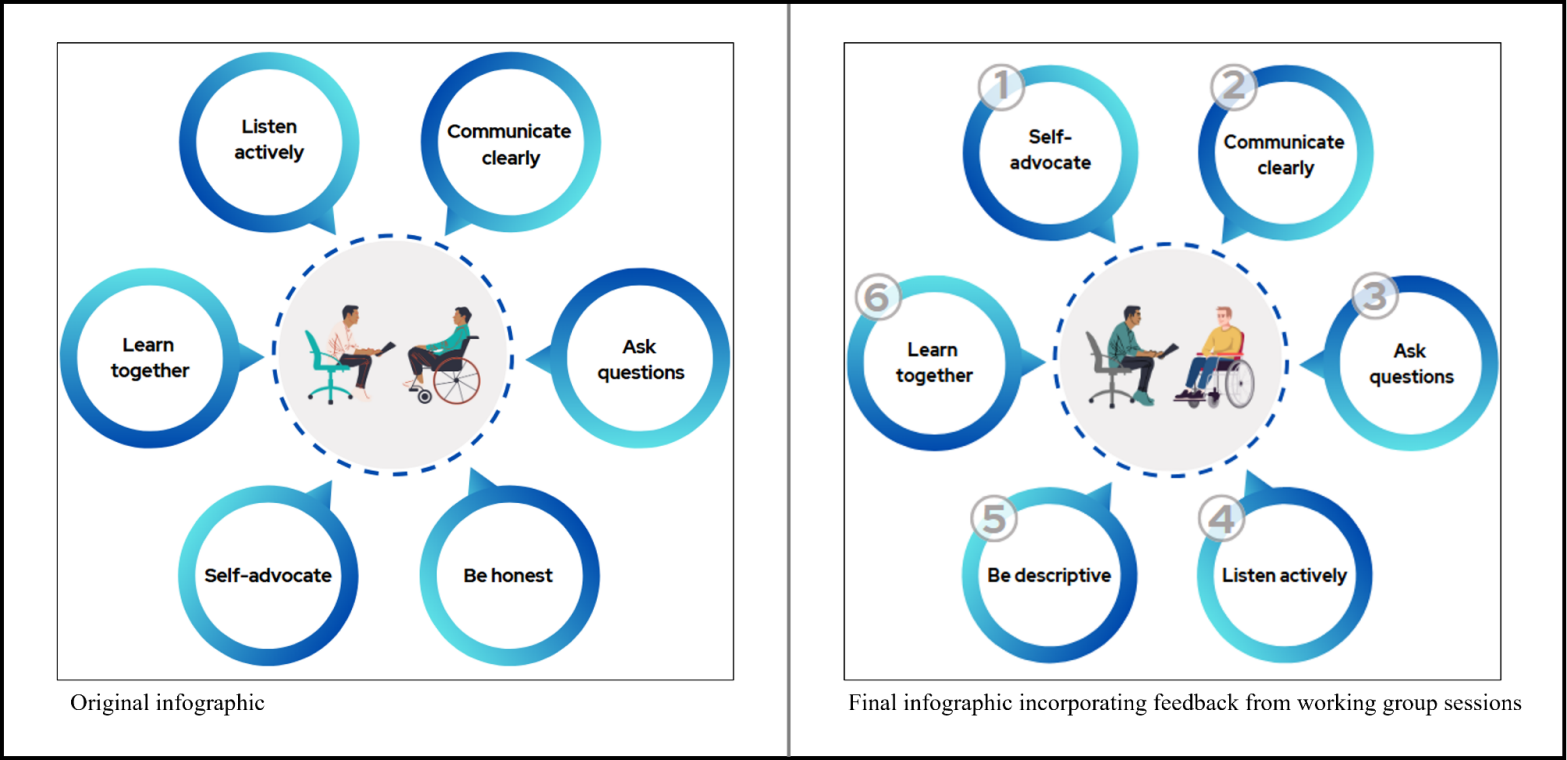
Original and final infographic based on the working group sessions

The feedback from Working Group Session 3 is presented in Table 2. The substantive feedback mapped onto five categories: first impressions, message and purpose, visual elements, layout and flow, and graphics. Overall, working group members were pleased with the visual content and variety of ways in which the information was being presented. They also offered a number of suggestions for improving the video and infographic that were presented. For the video, these recommendations included changing the artificial intelligence (AI)-generated voice used, slowing transitions between scenes, increasing the font size, using a larger, bolder, and cleaner-looking font, removing visuals that distracted from the message, and revising a scene with a patient-physician encounter to appear more patient-centred (e.g., physician sitting in front of their desk at eye-level with person with SCI/D, see Fig. 2). While participants agreed that they wanted a larger, bolder, and cleaner-looking font, when presented with five potential options, they had differing opinions on what fonts should be used for the headings and for the body text. We launched a poll to finalize the fonts for the toolkit. For the infographic, working group members recommended adding numbers to the circles that would correspond to the more in-depth written content, improving the colour contrast of the two characters in the middle circle, and changing the individual in the wheelchair to demonstrate better posture (see Fig. 3).

**Table 2:** Feedback obtained in working group session 3 and actions taken to address feedback

Feedback Category	Feedback	Actions Taken
First Impressions	• AI voice was not pleasant sounding (using AI is okay, but it needs to sound more human); some words were not pronounced clearly	• Identified four new AI voices to present to the group in the next meeting
	• The first slide disappeared too quickly and the words/voice over did not match (for the title)	• Extended the time on the title slide and ensured the words and voice over matched
	• Visual representation of the information was helpful	• No action required
Message and Purpose	• The message was clear and straightforward, but the timing needs to be longer to better get the message across	• Extended the time on the title slide to ensure viewers had adequate time to read
	• The AI voice was distracting from the video content being presented	• Identified four new AI voices to present to the group in the next meeting
Visual Elements (colours, typography, imagery)	• Colours were bland, contrast is important for readability of information on the slides (but avoid black on white), use solid colours rather than multiple shades	• Put together a colour scheme to present to the group for feedback, used solid colours and avoided shading, reviewed the colour contrast of the font on each background
	• Use larger, bolder, and cleaner font	• Identified potential fonts to present to the group in the next meeting
	• The logo in the bottom corner was distracting from the video content being presented	• Removed the logo by upgrading the software we used to create the video
	• Remove the blue border around the video	• Removed the blue border around the video
	• Remove the circle that encompasses the graphics to make icons bigger	• Removed the circle and enlarged the icons
	• Pictures in the background (on the wall in the office) were distracting	• Removed the pictures hanging on the wall in the scene
Layout and Flow	• The flow was good, but it would be helpful to have additional details that supplement the video	• Added written content to supplement the video
Graphics	• The patient looked nervous, consider changing her facial expression to look more relaxed	• Changed the patient’s facial expression to look more relaxed
	• The physician should be sitting (on rolling stool) at eye-level instead of standing over the patient	• Added a stool for the physician to be sitting at eye-level with the patient
	• Add a desk to the setting to make it look like the physician has come around her desk to talk with the patient	• Added a desk to the scene

*Abbreviations*: *AI* artificial intelligence

**Fig. 4:**
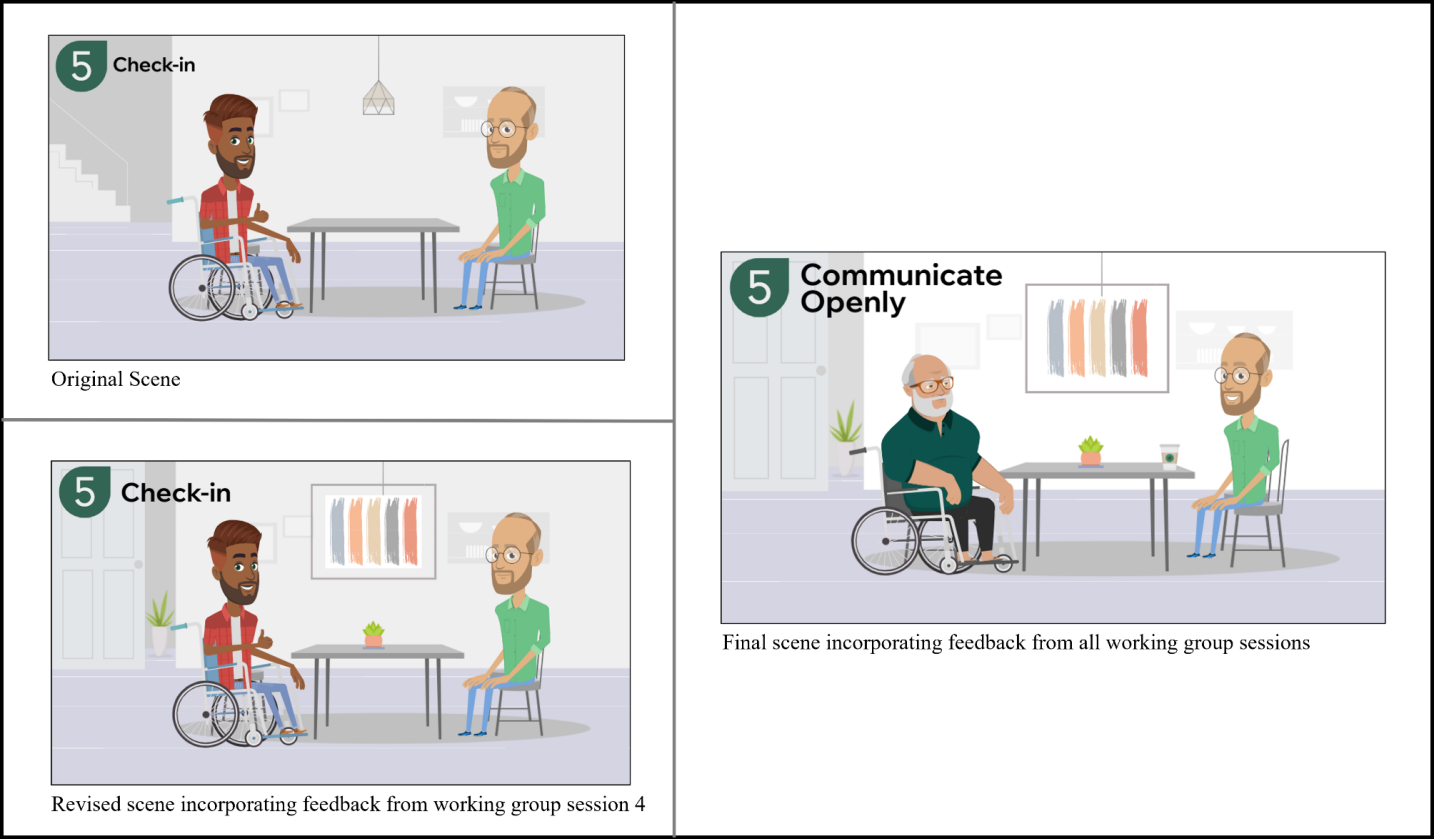
Original, revised, and final video scenes based on the working group sessions

The feedback from Working Group Session 4 is presented in Table 3. Similar to the previous session, feedback was categorized into five areas: AI voices, first impressions, visual elements, layout and flow, and graphics. Working group members selected two AI voices (one man and one woman) to be used in the videos. Participants liked the video animations and described how the information was presented in an accessible way. However, they identified the ending of the video as abrupt; as such, a summary was added to the end of the video. Overall, much of the feedback provided in this session was relatively minor and pertained to visual elements, such as changing colours to improve contrast, removing distracting icons and elements, changing facial expressions, and revising background scenery, which were easily addressed (see Fig. 4 for an example).

**Table 3:** Feedback obtained in working group session 4 and actions taken to address feedback

Feedback Category	Feedback	Actions Taken
AI Voices	• Avoid voices that have an Australian or British accent because we are targeting Canadians	• Selected two voices (man and woman) to be used in all videos
First Impressions	• Loved the animation and the software used	• No action required
	• The person with SCI/D had very small arms, could they be more muscular to reflect their use	• Unable to address due to limitations within the software for the original character; changed the character to address this feedback
	• Accessible and easy to understand, good for someone who is newly injured and may be more overwhelmed	• No action required
Visual Elements (colours, typography, imagery)	• Use one font style for headings and one for the body text	• Selected a font for headings and a font for the body text
	• It looked like the wheelchair handle was floating because of the low contrast with the background	• Increased the colour contrast with the background to make the entire wheelchair more visible
	• Some visual elements were distracting (e.g., speech bubbles with no text)	• Revised elements that were distracting (e.g., added text to speech bubbles to align with voice over)
	• Remove the speech bubble from the title slide	• Removed the speech bubble from the title slide and made the font larger
	• Liked how the scenes felt homey (e.g., use of plants, artwork), but recommended adding these throughout	• Added elements throughout the video to make the scenes feel like a home
	• One scene had stairs in the background, recommend changing this scene or adding a stair lift	• Revised the scene to remove the stairs in the background, as a stairlift could not be added due to software limitations
Layout and Flow	• Remove the slide of the individual with SCI/D thinking, as it is unclear what he is thinking about and it is distracting from the messaging	• Removed the slide
	• The ending is abrupt	• Added a summary slide at the end of the video to recap the information
Graphics	• Uncross the arms of the caregiver on the title slide and move the people closer together	• Revised the title slide to address
	• One icon of a woman did not have a face, did not like the ‘faceless woman’	• Revised the icon to include one of a woman with a face
	• Increase the size of the icons to be able to clearly see what they are	• Increased the size of the icons

Table 4 displays how the concept mapping clusters mapped onto the toolkit modules and provides a brief overview of the objective of each module. The working group thought the organization and objectives of the modules were clear. Members liked the variety of content being created and talked about the benefit of having visuals to supplement the written content. Members liked the overall colour scheme. Some recommended adding a few more ‘pops’ of colour; however, not all members agreed with this. We presented the working group with five potential names for the toolkit and through small and large group discussions, MedManageSCI was selected, as participants reflected on the importance of the name including reference to both medications and SCI/D. Through an iterative process with the website development company, an online version of MedManageSCI was created.

**Table 4:** Concept mapping clusters, toolkit modules, and module objectives

Concept Mapping Cluster	Modules	Objective of Module
Not applicable	Module 1: Background Information about Medication Self-Management	Brief overview of what medication self-management is and what information individuals can expect to find in the toolkit
Cluster 7: General medication information	Module 2: General Medication Education and Awareness	Provide general information about medications, healthcare providers, and where to access information
Cluster 1: Information-sharing and communication	Module 3: Reflections and Advocacy	Provide information about self-reflecting on medications and self-advocating for what’s important
Cluster 1: Information-sharing and communication Cluster 2: Healthcare provider interactions and involvement	Module 4: Communication and Information Sharing	Provide questions and topics to discuss with healthcare providers; strategies for communicating and building relationships
Cluster 8: Practical information and strategies related to medication-taking	Module 5: Practical Tips and Strategies for Medication Management	Provide practical tips for medication management to support medication-taking and lifestyle considerations
Cluster 6: Safety and lifestyle considerations	Module 6: Medication Safety and Management of Side Effects	Provide information on medication safety, including changes to regimen, side effects, dependency, and pain
Cluster 4: Supports and services for accessing prescription medications and medication information Cluster 5: Information on non-prescription medication and medication supplies	Module 7: Access to Medications, Supplies, and Services	Provide information on financial considerations, access to specific medications, and services to support medication management
Cluster 3: Peer and community connections	Module 8: Peer Connections and Support	Provide information and strategies for creating peer connections
Cluster 8: Practical information and strategies related to medication-taking	Module 9: Managing Expectations and Adapting to Change	Provide information about how to manage changes with medication self-management post-injury and over time, as well as strategies for coping with medication-taking

### Stage 3: test

#### Working group sessions

The feedback from Working Group Session 6 is presented in Table 5. Participants provided recommendations to improve the website’s functionality and make navigation more intuitive by adding a ‘Home’ button to the toolbar. There was consensus from working group members that some of the pictures used throughout the toolkit needed to be changed to be more reflective of the SCI/D population by including more up-to-date wheelchairs. Through discussion, it was decided that the information on the ‘About’ page would contain a headshot, first name, level of injury, and year of injury for individuals with SCI/D. As for healthcare providers, a headshot, first name, and profession were included. Caregiver working group members chose not to be profiled. Lastly, the working group talked about liking the testimonials section on the home page, but emphasized the importance of expanding on this by having a feature that allowed other users to provide a testimonial or feedback.

**Table 5:** Feedback obtained in working group session 6 and actions taken to address feedback

Website Page	Feedback	Actions Taken
All	• Functionality: Include a ‘home’ button in the toolbar so navigation to the home page is clear (rather than having to click on the logo)	• Added a ‘home’ button to the toolbar
All	• Images: Update some of the photographs to reflect more up-to-date wheelchairs	• Updated some of the stock images to reflect more up-to-date wheelchairs
Home	• Testimonials: Include testimonials from others with SCI/D about their reflections on the toolkit, include a comment feature to collect testimonials on an ongoing basis	• Collected testimonials from the working group to include on the home page and added a comment feature to collect ongoing feedback from users
About	• Working group details: include picture, first name, level of injury, and year of injury for individuals with SCI/D and include picture, first name, and profession for providers	• Added the recommended details for all members of the working group

## Discussion

The prototype of MedManageSCI, a toolkit to promote medication self-management among adults with SCI/D, was co-designed with adults with SCI/D, caregivers, and healthcare providers. To our knowledge, this is the first toolkit co-designed with end-users and key interest groups that comprehensively addresses medication self-management following SCI/D. Following the Good Things Foundation Pathfinder Model as a guide [34], the prototype of the toolkit was created through a three-stage process of understanding and defining end-user and key interest group needs, generating ideas and developing the toolkit prototype, and testing the toolkit. While the benefits of co-design have been previously described [28–31], this comprehensive process offered a number of benefits, specifically its adaptability and use of engagement methods that supported relationship-building.

Our participants and working group members were involved in generating the content areas, providing feedback on the written and visual content, deciding on a name for the toolkit, and providing initial feedback on the website’s functionality and navigation. Overall, participants were pleased with the variety and depth of content that was created and enjoyed having visual content to supplement the written content. Specific recommendations were made to enhance the content, which included improving the message and purpose, visual elements, layout and flow, and graphics. Similar recommendations were identified in Leung et al.’s co-development of a digital toolkit to promote self-management behaviours among individuals with systematic lupus, where their users provided feedback to improve the resource content, formatting, and language [42]. In terms of actions taken to address participants’ feedback, we followed a similar process to other co-design studies [42–44], where feedback was categorized, supported with evidence, and specific actions taken to address the feedback were outlined.

The co-design approach was critical in identifying key revisions that may have impacted the end-users’ perceptions of the toolkit and its content. For example, in the original layout of the website, the navigation toolbar did not contain a “Home” button. Working group members expressed the importance of adding one to make navigation of the website clear and simple. This demonstrates the importance of the working group’s involvement throughout the co-design process. As highlighted by Thorburn et al., co-design supports a collective, knowledge-generating process that supports the creation of new knowledge [45]. Members of our working group brought contextual and practical knowledge through their lived experience, which diversified the feedback and enhanced the MedManageSCI prototype. In terms of visual content, the working group commented on ensuring the videos were portraying patient-centred interactions between physicians and persons with SCI/D. To do so, the working group recommended that the physician was sitting in front of their desk and at eye-level with the person with SCI/D. Additionally, the working group made recommendations to ensure the scenes of the home environment were representative of the SCI/D population. For example, removing stairs or adding stair lifts. Without the co-design process, website navigation may have been more challenging for the end-users, visual content may not have portrayed positive patient-physician encounters, and video scenes may not have displayed accessible home environments. Ultimately, through co-design, these recommendations assisted in improving the relevance and applicability of MedManageSCI to the end-users [28, 29].

The Good Things Foundation Pathfinder Model offered flexibility in the process, with stages being adaptable based on context [34]. The flexibility of the Model was beneficial. To explain this further, some co-design methods encourage in-person engagement with end-users to promote more authentic engagement and build rapport. However, in-person engagement is not always feasible due to accessibility challenges, financial constraints, personal or family health, and travel considerations [46], especially for persons with SCI/D who may also experience mobility challenges and secondary health conditions [47, 48]. The Good Things Foundation Pathfinder Model focused more on what was being accomplished rather than how it was being done, which allowed us to run our co-design sessions virtually. Not only did this facilitate the involvement of persons with SCI/D, but it also made it more feasible to include participants from a much larger geographic area, which for this study included individuals from across Canada. The benefit of using technology to more feasibly include participants from a larger geographical area has been widely noted in the literature [49]. For example, Kennedy and colleagues described translating a face-to-face co-design portion of project to online during the COVID- 19 pandemic [49]. In doing so, the authors reflected on how the virtual delivery allowed for more equitable representation of individuals regardless of their geographical location as it ensured that individuals who participated had a desire to be involved, rather than simply having the means to do so. Despite the possibility of including individuals from a larger geographic region, the use of technology to conduct virtual co-design sessions may also exclude individuals, including those without access to internet or technology and those who are unable or uncomfortable using technology. As such, when conducting co-design sessions, it is important to not only identify who is present, but also who may be excluded based on the selected method of engagement.

The use of technology to conduct virtual co-design sessions has been previously reported both within and outside the SCI/D population [49–53]. To support successful virtual co-design, the use of breakout rooms has been recommended to facilitate collaborative discussion [49] and was a notable strength when conducting our sessions. In our sessions with the full working group, we used breakout rooms for activities to allow more thorough discussion. This allowed individuals more time to share their thoughts and engage in a more detailed discussion, which contributed to more data being generated [49]. The use of breakout rooms has the potential to help build rapport among members involved in the co-design, which can increase their comfort and improve overall engagement [49, 54]. We found that rapport and trusting relationships between the research team and members of the working group were rapidly built using this approach. This was a key strength of this study because members of the working group were able to provide constructive feedback, which was critical in improving the overall applicability, design, and function of the toolkit. Ultimately, following the Good Things Foundation Pathfinder Model as a guide allowed us to meaningfully engage with end-users and key interest groups throughout the design process.

While the involvement of end-users and key interest groups was beneficial throughout the planning and design process, other scholars have reflected on the importance of patient and public involvement as part of the implementation process as well [55–57]. Voorheis and colleagues conducted a descriptive qualitative study to understand how to maximize patient and public involvement in the design of digital health interventions [55]. In doing so, they identified that patient and public partners provided key feedback on multiple design features, including the interface, user experience, behaviour change, and integration with existing supports. However, in addition to this, the authors also found that participants desired greater involvement in the digital health intervention’s implementation, as they felt as though they were able to provide insight into how their population would perceive their needs as well as seek, reach, and access the intervention. Thus, Voorheis and colleagues recommend involving partners beyond the design stage to optimize the intervention’s sustainability. In doing so, this allows for ongoing adaptation to the digital health solution to ensure it is meeting the end-users’ needs. Similarly, in a Patient Engagement in Health Implementation Research Logic Model developed by Bisson et al. [57], the authors discussed how the involvement of partners can improve intervention implementation by ensuring it reflects end-users’ priorities and can facilitate the establishment of a trusting relationship between the research team and their partners. While co-design during implementation was not part of the Good Things Foundation Pathfinder Model, it is an important consideration as we move this work forward and prepare for implementation.

Barriers to intervention and tool implementation are well-documented and may include micro, meso, and macro-level factors [58]. Specific to the implementation of MedManageSCI, we may face barriers such as awareness of the toolkit, perceived applicability, inability to adapt or personalize the toolkit, adoption from adults with SCI/D, caregivers, healthcare providers, and relevant organizations, and support from organizations with dissemination, all of which may also impact sustainability. To support the implementation of MedManageSCI, a framework, such as the Consolidated Framework for Implementation Science [59], could be used to tailor our plan by identifying mitigation strategies that correspond to the barriers. In doing so, we will support adoption and improve the likelihood of sustainability over time.

### Future research

MedManageSCI has the potential to fill a gap and provide adults with SCI/D with a comprehensive resource to assist with the self-management of medications. Further research is needed to finalize the toolkit by ensuring the content is understandable and accessible to the end-users. Given that this is a self-management toolkit, it is designed to be used by the end-users; however, more work is needed to better understand if it should be used in conjunction with caregivers and healthcare providers. Additionally, a mixed-methods pilot test will be conducted to assess the feasibility, acceptability, appropriateness, and usability of MedManageSCI. Outcomes related to medication self-management will also be assessed, including beliefs about medications, medication management capacity, medication self-efficacy, and quality of life. To assess the overall impact of the MedManageSCI toolkit, it will be key to examine if it improves medication management capacity, medication self-efficacy, and quality of life among adults with SCI/D. It will be important to understand the frequency of use, as well as how and why individuals are using the toolkit.

### Limitations

There are a few limitations to note. All sessions were conducted in English, with individuals who were comfortable reading and communicating this language. Additionally, the toolkit, including all written and visual content, was developed in English. Given the diverse population of Canada, future work could explore translating the toolkit across different languages. Despite attempts to include individuals from across Canada, only two provinces were represented in our working group, Ontario and Alberta, so it is possible that this sample is not representative of the SCI/D population across Canada. As we continue with this work, it would be beneficial to recruit representatives across more Canadian provinces. All sessions were conducted virtually, so while this aided in involving participants from across Canada and minimized accessibility challenges, it also may have excluded some interested individuals. Despite attempts to recruit more caregivers for the Working Group, we were only able to have ongoing involvement from one caregiver; therefore, it is possible that the results are not reflective of caregivers across Canada. Lastly, we did not formally measure the working groups’ perceptions of engagement. As we move forward with this research, it will be important to better understand the working groups’ experiences with the process and how it can be improved.

## Conclusions

MedManageSCI is a toolkit that aims to improve medication self-management among adults with SCI/D. The co-design process used to guide the development of the MedManageSCI prototype offered numerous benefits, including: flexibility across the model stages and promotion of relationship development between the research team and end-users. Future work includes refining the content and pilot testing the toolkit to assess its feasibility, acceptability, and appropriateness, along with outcomes related to medication self-management.

## Data Availability

All relevant data are within the manuscript.

## Abbreviations

AI: Artificial intelligence
SCI/D: Spinal cord injury/dysfunction
